# Polyethylene glycol combined with lactulose has better efficacy than polyethylene glycol alone in bowel preparation before colonoscopy: A meta-analysis

**DOI:** 10.1016/j.clinsp.2023.100172

**Published:** 2023-04-03

**Authors:** Xiaofen Zhang, Yishu Chen, Ye Chen, Wei Zhu, Chenxi Tang, Shelby Lamm, Lan Li

**Affiliations:** a Department of Gastroenterology, The First Affiliated Hospital, Zhejiang University School of Medicine, Zhejiang Province, China; b Department of Mathematics and Statistics, Northern Arizona University, Flagstaff, USA

**Keywords:** Bowel preparation, Colonoscopy, Polyethylene glycol, Lactulose, Meta-analysis

## Abstract

**Background:**

The accuracy of diagnosis and the safety of treatment in colonoscopy depends largely on the quality of bowel cleansing. This study aimed to compare the efficacy and adverse reactions of Polyethylene Glycol (PEG) combined with lactulose with that of PEG alone in bowel preparation before colonoscopy.

**Methods:**

The authors searched a number of databases including EMBASE, MEDLINE, Cochrane Library, and China Academic Journals Full-text Database. The authors screened according to literature inclusion and exclusion criteria, assessed the quality of the included literature, and extracted the data. The meta-analysis of included literature used RevMan 5.3 and Stata 14.0 software.

**Results:**

A total of 18 studies, including 2274 patients, were enrolled. The meta-analysis showed that PEG combined with lactulose had a better efficacy (OR = 3.87, 95% CI 3.07‒4.87, p = 0.000, and *I^2^* = 36.2% in the efficiency group; WMD = 0.86, 95% CI 0.69‒1.03, p = 0.032 and *I^2^* = 0% in the BBPS score group) in bowel preparation for patients with or without constipation. Moreover, PEG combined with lactulose had fewer adverse reactions, including abdominal pain (OR = 1.42, 95% CI 0.94‒2.14, p = 0.094), nausea (OR = 1.60, 95% CI 1.13‒2.28, p = 0.009) and vomiting (OR = 1.77, 95% CI 1.14‒2.74, p = 0.011), than PEG alone. No significant reduction in the incidence of abdominal distention was observed.

**Conclusion:**

PEG combined with lactulose may be a better choice for bowel preparation before colonoscopy compared with PEG alone.

## Highlights

• High quality of bowel cleansing is important to the accuracy of diagnosis and the safety of treatment in colonoscopy.
• PEG combined with lactulose has a better efficacy in bowel preparation than PEG alone.
• PEG combined with lactulose has fewer adverse reactions than PEG alone in bowel preparation.

## Introduction

With the development of endoscopic technology, colonoscopy has been widely utilized in the diagnosis and treatment of intestinal diseases. The accuracy of diagnosis and the safety of treatment in colonoscopy depends largely on the quality of bowel cleansing.^1^ Inadequate bowel preparation may prolong the operating time, increase risks of endoscopy and shorten the interval of follow-up.^2^ Additionally, insufficient intestinal preparation may lower intestinal adenoma detection rate due to a significantly higher rate of missed diagnosis.^3^ It is reported that twenty to twenty-five percent of colonoscopy bowel preparation cases were far from satisfactory.^2^ Polyethylene Glycol (PEG) is the most commonly used intestinal cleanser in bowel preparation, however, some studies showed that PEG might induce symptoms of intolerance, such as nausea, bloating, palpitations and dizziness. In addition, the cleansing effect turned out to be unstable and might vary between individuals.^4^ To address this problem, several studies have evaluated various combinations of two agents to improve compliance and reduce adverse events, including the addition of laxatives to the PEG solution or a combination of two types of laxatives, such as lactulose and magnesium sulfate.^5^

Lactulose oral solution passes unabsorbed down to the colon, where lactulose shows the osmotic activity as a disaccharide. It retains water and electrolytes in the intestinal cavity and produces a hyperosmotic effect, resulting in the excretion of intestinal ammonia and other toxins. It is not only quite palatable with few adverse effects but conducive to relieving constipation symptoms.^6^ Recently, some studies have adopted lactulose as an intestinal cleanser, which exhibited a positive effect.^7^, ^8^, ^9^

However, it is still unknown whether PEG combined with lactulose serves as a better solution for bowel preparation than PEG alone since previous studies showed conflicting results. The aim of this meta-analysis was to systematically evaluate the efficacy, as well as adverse reactions, of PEG combined with lactulose compared with PEG alone for bowel preparation before colonoscopy in adults to provide a new methodology for clinical practice.

## Materials and methods

### Inclusion and exclusion criteria

Eligible studies included in this meta‐analysis should meet all the following criteria: (a) Randomized controlled studies involving more than ten adult patients (age ≥ 18 years) who underwent a colonoscopy and used PEG with or without lactulose for bowel preparation; (b) Studies with data on patients having constipation or not; (c) Studies that made a clear evaluation on the efficacy of bowel preparation with or without a detailed description of adverse reactions.

### Search strategies

The authors searched the databases (EMBASE, MEDLINE, Cochrane library and China Academic Journals Full-text Database) for studies published by October 2022 with the following terms: Colonoscopy AND (Macrogol OR PEG OR Polyethylene OR Polyethylene Oxide OR Oxide, Polyethylene OR Oxides, Polyethylene OR Polyethylene Oxides OR Polyethyleneoxide OR Polyethyleneoxides OR Polyoxyethylenes OR Polyoxyethylene OR Polyglycol OR Polyglycols OR Glycol, Polyethylene OR Glycols, Polyethylene OR Carbowax) AND (Lactulose OR Duphalac OR Normase OR Amivalex) AND (Cathartics OR Bowel Evacuants OR Evacuants, Bowel OR Purgatives OR Bowel Preparation Solutions OR Preparation Solutions, Bowel OR Solutions, Bowel Preparation).

### Data extraction

The studies were initially scrutinized by two reviewers independently. First, titles and abstracts were read carefully, and those that apparently did not meet the inclusion criteria were excluded. Then, the full text was further evaluated to determine eligibility. Finally, information of the included studies (title, author, year of publication, sample size, intervention measures, observation indicators, adverse reactions, etc.) was recorded according to the pre-established data extraction table. The records were later rechecked by the other reviewer. Disagreements were addressed through discussion or with the help from a third party.

### Quality evaluation

The Cochrane risk-of-bias tool was utilized for evaluating the quality of the included studies.^10^ The evaluation was conducted from the following aspects: random sequence generation, allocation concealment, blinding in the experimental design, completeness of outcome data, presence of selective reporting and other biases.

### Statistical analysis

Analysis of the extracted data was performed with RevMan 5.3 and Stata 14 software. Odds Ratio (OR) was calculated for categorical data and Weighted Mean Difference (WMD) for qualitative data, both represented by 95% Confidence Interval (95% CI). Pooled estimates were obtained using the fixed‐model (Mantel and Haenszel) method (if *I^2^* ≤ 50%, p > 0.1) or random‐model (M‐H heterology) method (if *I^2^* > 50%, p ≤ 0.1).^11^ Statistical heterogeneity was assessed by Cochran's Q test and the I^2^ statistic. Meta‐regression was used to detect sources of heterogeneity (Monte Carlo permutation test). Results are listed in the forest chart, and the publication bias is represented by the inverted funnel chart.

Studies scored the quality of bowel cleansing using either the Ottawa Bowel Preparation Quality Scale (OBPS), Boston Bowel Preparation Scale (BBPS), or their own non-validated scales. For studies using the OBPS, a total score of 6 or lower was deemed adequate. For studies using the BBPS, a total score of 6 or higher was deemed adequate. For studies not using a validated scale, their scale's judgment of adequate and inadequate was used.^12^

To access the probability and confidence interval of a random sample drawn from the data source that has the Gaussian distribution with mean and standard distribution and is over (BBPS) or below (OBPS) the threshold 6, the authors did the following. We first simulated data sets with the same mean, standard distribution, and sample size. Then, we computed the ratio of samples that is over (BBPS) or below (OBPS) the threshold 6 for each simulation. Lastly, the authors summarized the simulations and found the mean and 95% CI.

## Results

### Literature search

**Fig. 1:**
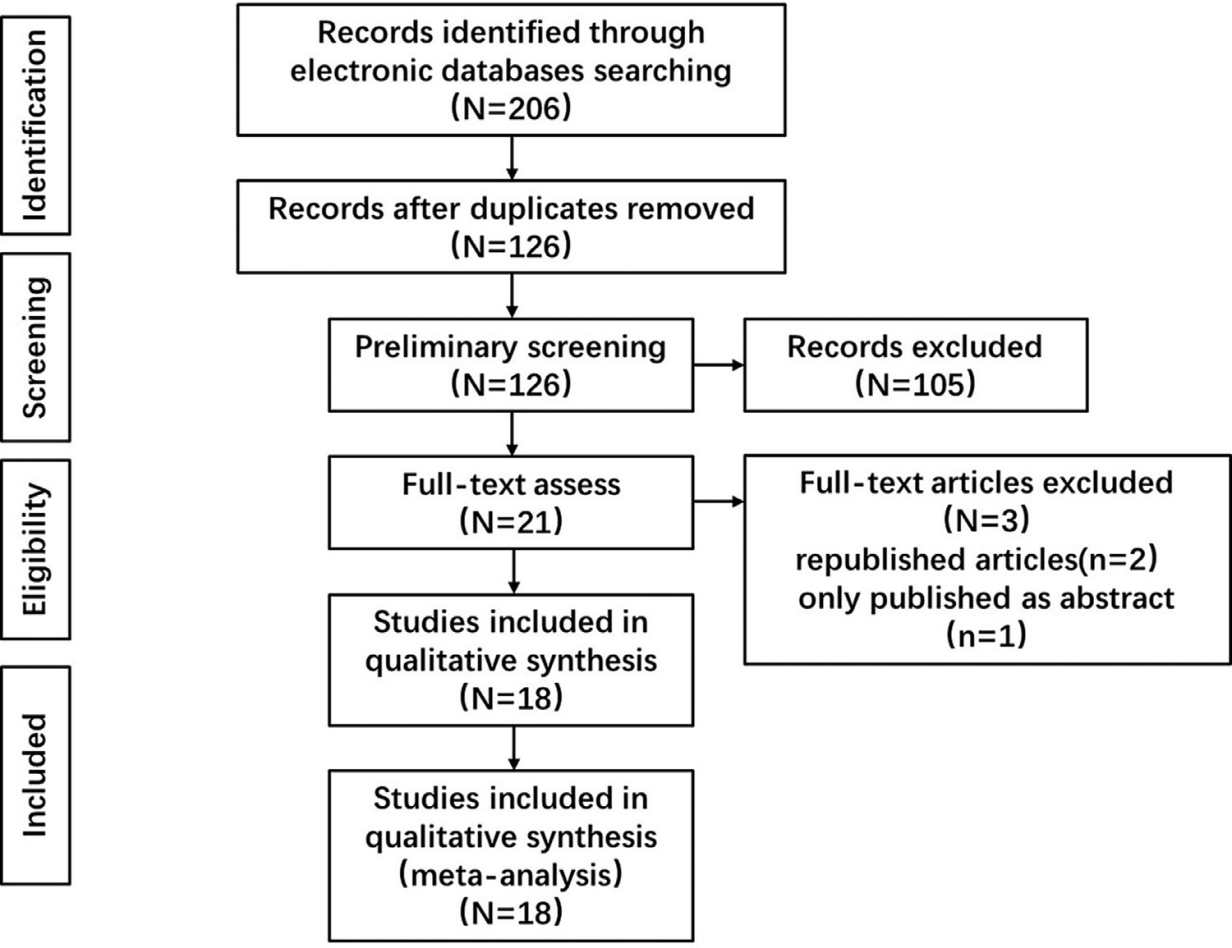
Flow chart of study selection.

A total of 206 relevant documents were obtained using the above-stated search strategy. After removing duplicates, 126 documents remained. Those documents were further screened and removed if they did not meet the inclusion criteria. Then, 21 full texts were assessed, after which 2 republished articles and 1 article with an abstract only were excluded. 18 articles^13^, ^14^, ^15^, ^16^, ^17^, ^18^, ^19^, ^20^, ^21^, ^22^, ^23^, ^24^, ^25^, ^26^, ^27^, ^28^, ^29^, ^30^ were included in the exercise performance (16 in Chinese and 2 in English). The flow chart of the literature screening is shown in Fig. 1.

A total of 206 relevant documents were obtained using the above-stated search strategy. After removing duplicates, 126 documents remained. Those documents were further screened, reviewed, and further assessed using the inclusion criteria, resulting in 18 articles^13^, ^14^, ^15^, ^16^, ^17^, ^18^, ^19^, ^20^, ^21^, ^22^, ^23^, ^24^, ^25^, ^26^, ^27^, ^28^, ^29^, ^30^ included in the exercise performance (16 in Chinese and 2 in English). The flow chart of the literature screening is shown in Fig. 1.

### Basic characteristics

Basic characteristics of the included studies, all of which were published in the last 7 years (2015–2021), are shown in Table 1. There are 2518 patients enrolled in total, including 1255 in the test group (PEG combined with lactulose) and 1263 in the control group (PEG alone). Of the included studies, 13^16^, ^17^, ^18^, ^19^, ^20^, ^21^^,^^23^^,^^24^^,^^27^, ^28^, ^29^, ^30^ reported data on constipation, while the other 5^13^, ^14^, ^15^^,^^23^^,^^24^ did not. The evaluation method of bowel preparation quality varied between studies. Most adopted BBPS, while some used a non-validated scale, and 2 studies^28^^,^^29^ used OBPS.

**Table 1:** Characteristics of studies included in meta-analysis.

**Study (year)**	**PEG-lactulose (n)**	**PEG (n)**	**Treatment**	**Design**	**Bowel assessment**	**With constipation**
Hu et al. (2020)	180	180	PEG 2L+lactulose 100 mL	Double-blind, randomised	BBPS	No
			PEG 2L			
Song WX et al. (2019)	149	151	PEG 2L+lactulose 90 mL	Randomised	BBPS	No
			PEG 2L			
Liu FX et al. (2015)	61	60	PEG 2L+lactulose 50 mL	Randomised	BBPS	No
			PEG 2L			
Zhang ZY et al. (2018)	16	16	PEG 2L+lactulose 90 mL	Randomised	BBPS	Yes
			PEG 2L			
Zheng Y et al. (2018)	40	40	PEG 2L+lactulose 60 mL	Randomised	BBPS	Yes
			PEG 2L			
Yu ZB et al. (2018)	36	36	PEG 2L+lactulose 120 mL	Single-blind, randomised	BBPS	Yes
			PEG 2L			
Nong CS et al. (2015)	36	36	PEG 1L+lactulose 20 mL	Randomised	Non-validated scale	Yes
			PEG 1L			
Wu J et al. (2018)	84	91	PEG 3L+lactulose 180 mL	Randomised	BBPS	Yes
			PEG 3L			
Zhang XT et al. (2019)	45	45	PEG 4L+lactulose 60‒180mL	Randomised	BBPS	Yes
			PEG 4L			
Xu HR et al. (2015)	90	90	PEG 0.75L+lactulose 90 mL	Single-blind, Randomised	BBPS	Yes
			PEG 0.75L			
Yang J et al. (2016)	50	50	PEG 2.5L+lactulose 20 mL	Randomised	Non-validated scale	No
			PEG 3L			
Jiang XL et al (2017)	50	50	PEG 1-2L+lactulose 90 mL	Single-blind, randomised	BBPS	Yes
			PEG 2‒3L			
Wang Q et al. (2015)	74	74	PEG 2L+lactulose 120 mL	Randomised	Non-validated scale	Yes
			PEG 2L			
Hu XB et al. (2020)	42	42	PEG 2L+lactulose 120 mL	Randomised	BBPS	No
			PEG 2L			
Huang RW et al. (2015)	45	45	PEG 2L+lactulose 60 mL	Randomised	Non-validated scale	Yes
			PEG 3L			
Wu Y et al. (2016)	53	53	PEG 2L+lactulose 30 mL	Randomised	OBPS	Yes
			PEG 2L			
Lu et al. (2016)	45	45	PEG 2L+lactulose 30 mL	Single-blind, randomised	OBPS	Yes
			PEG 2L			
Han LX et al. (2016)	35	35	PEG 3L+lactulose 45 mL	Randomised	Non-validated scale	Yes
			PEG 3L			

### Risk of bias

**Fig. 2:**
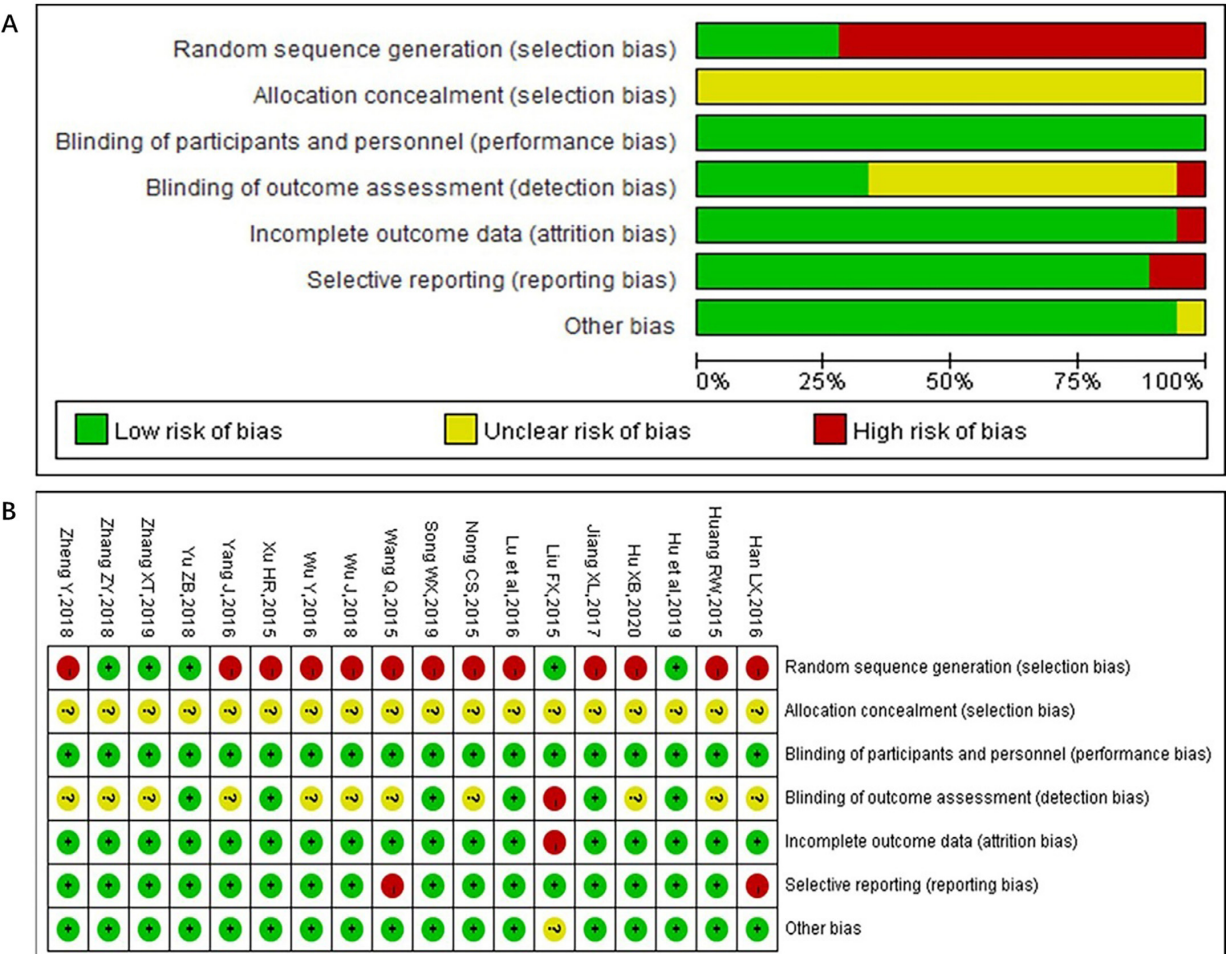
Risk of bias graph and summary. (A) Risk of bias graph. (B) Risk of bias summary.

Of the 18 included studies, 5 were grouped by a random number table and 13 mentioned randomizations but did not give a detailed description. 6 of the 20 studies clearly described blinding methods in endoscopy and intestinal cleanliness assessment, while the remaining ones were ambiguous. Allocation concealment was ‘unclear’ in all studies. The risk of bias graph and summary is shown in Fig. 2.

### Quality of bowel preparation

**Fig. 3:**
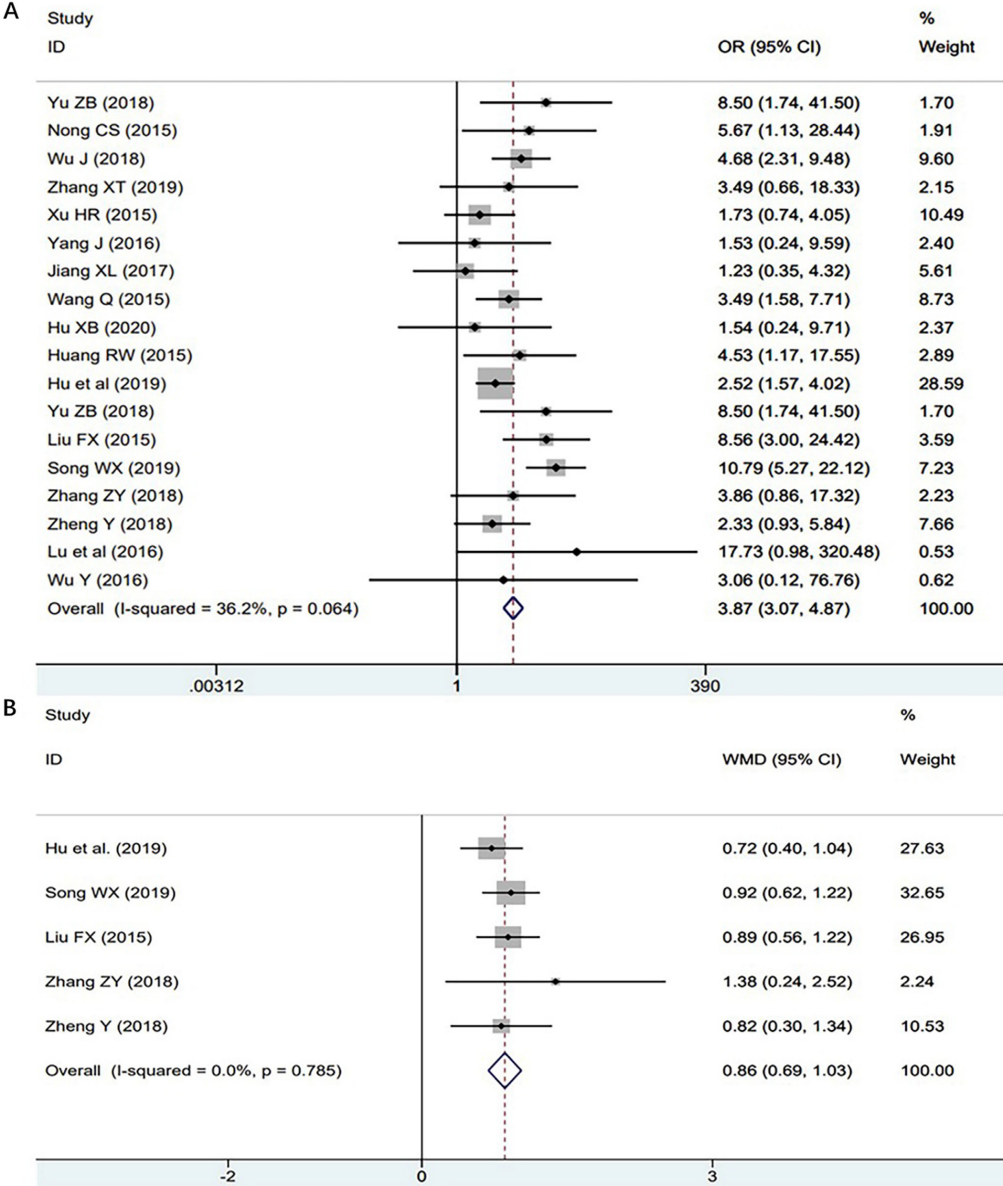
Forest plot of bowel preparation quality. (A) Forest plot of bowel preparation quality assessed with efficiency. (B) Forest plot of bowel preparation quality assessed with BBPS score.

The authors assessed the quality of bowel preparation using efficiency and BBPS score respectively. All studies^13^, ^14^, ^15^, ^16^, ^17^, ^18^, ^19^, ^20^, ^21^, ^22^, ^23^, ^24^, ^25^, ^26^, ^27^, ^28^, ^29^, ^30^ were included in the efficiency group, while only 5 studies^13^, ^14^, ^15^, ^16^, ^17^ provided detailed BBPS scores. The analysis highlighted a significantly higher overall cleansing success rate for patients receiving PEG combined with lactulose than PEG alone (OR = 3.87, 95% CI 3.07‒4.87, p = 0.000, and *I^2^* = 36.2% in the efficiency group; WMD = 0.86, 95% CI 0.69‒1.03, p = 0.032 and *I^2^* = 0% in the BBPS score group). The forest plot of bowel preparation quality is shown in Fig. 3.

**Fig. 4:**
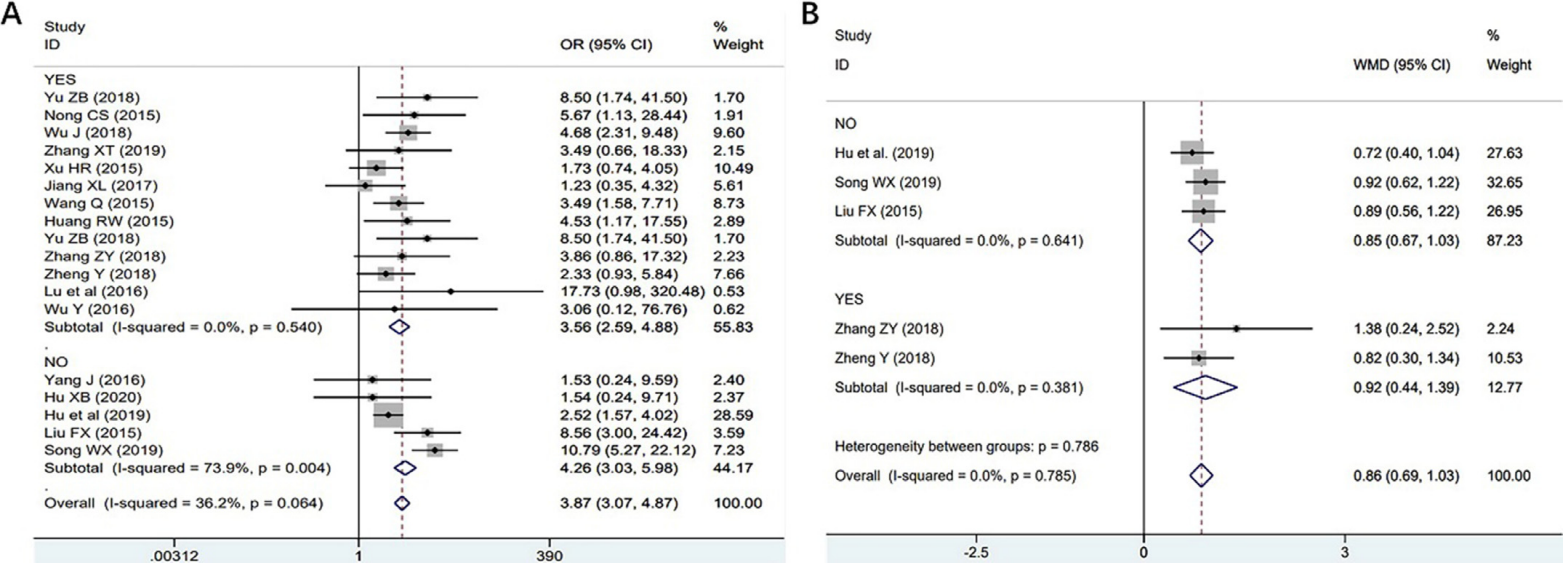
Forest plot of bowel preparation quality in subgroup analysis. (A) Forest plot of bowel preparation quality assessed with efficiency in subgroups. (B) Forest plot of bowel preparation quality assessed with score in subgroups.

Next, the authors determined whether PEG combined with lactulose also worked better for patients with constipation than PEG alone. The authors conducted subgroup analysis of patients with or without constipation. In the efficiency group, 13 studies^16^, ^17^, ^18^, ^19^, ^20^, ^21^^,^^24^^,^^25^^,^^27^, ^28^, ^29^, ^30^ with constipation and 5 studies^13^, ^14^, ^15^^,^^23^^,^^24^ without constipation. The results showed that PEG combined with lactulose was better than PEG alone for bowel preparation in the constipation subgroup (OR = 3.56, 95% CI 2.59‒4.88, p = 0.000). There was low heterogeneity (*I^2^* = 0%) between the studies. The subgroup without constipation also showed better efficiency in PEG combined with the lactulose group (OR = 4.26, 95% CI 3.03‒5.98, p = 0.000), but there was high heterogeneity (*I^2^* = 73.9%) between the studies. (Fig. A). In the BBPS score group, 2 studies^16^^,^^17^ provided data on constipation while the other 3^13-15^ did not. The results showed that PEG combined with lactulose was better than PEG alone for bowel preparation both in the constipation subgroup (WMD = 0.85, 95% CI 0.67‒1.03, p = 0.000) and in the subgroup without constipation (WMD = 0.92, 95% CI 0.44‒1.39, p = 0.000) (Fig. 4B).

### Adverse reactions

The adverse reactions after drug administration largely determine a patient's compliance, which in turn affects the drug efficacy, especially in bowel preparation. The main adverse reactions after bowel cleanser administration include abdominal pain, abdominal distention, nausea, and vomiting. In this study, the authors compared the adverse reactions of PEG combined with lactulose and PEG alone.

### Abdominal pain

**Fig. 5:**
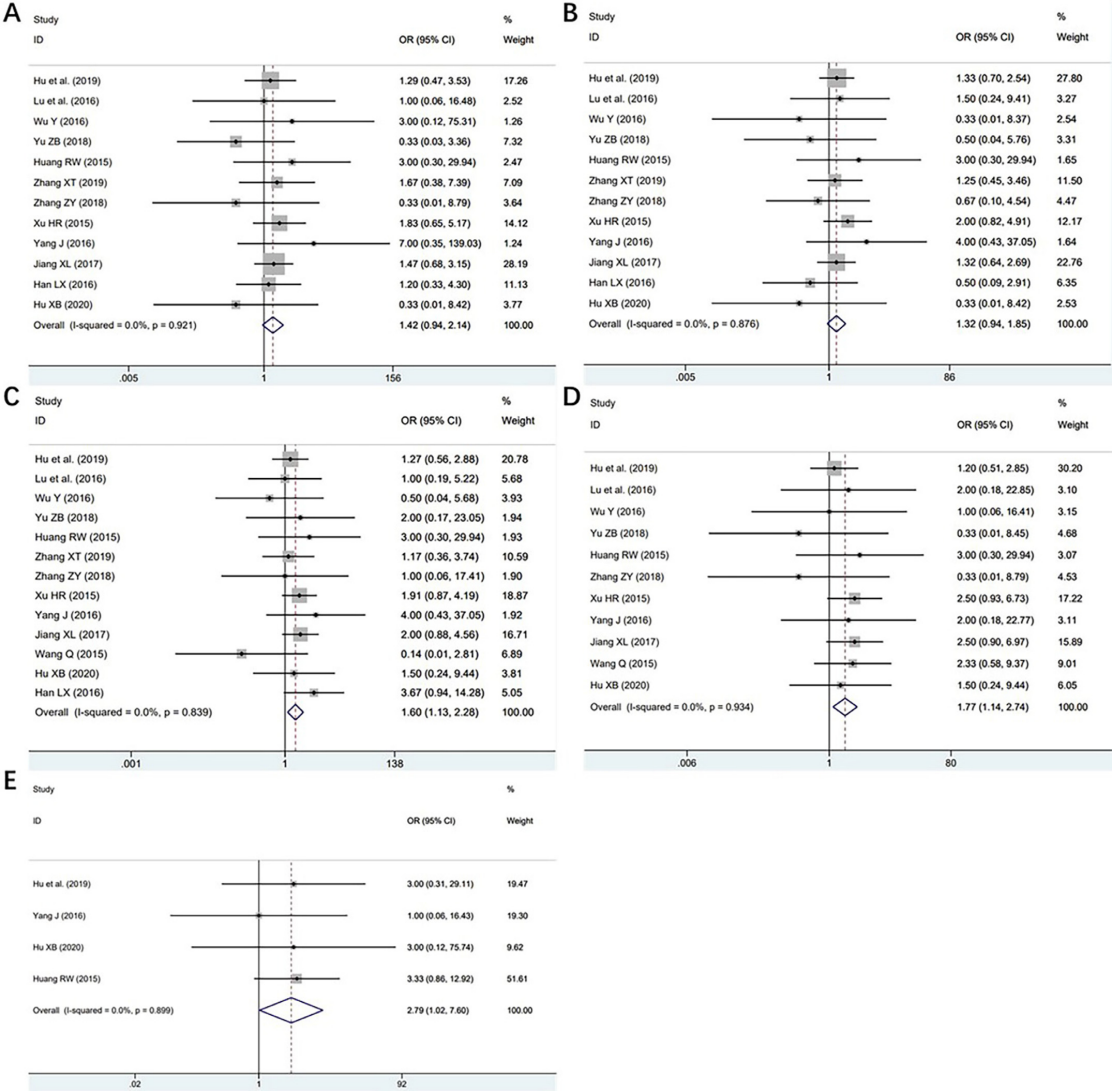
Forest plot of adverse reaction. (A) Forest plot of abdominal pain. (B) Forest plot of abdominal distention. (C) Forest plot of nausea. (D) Forest plot of vomiting. (E) Forest plot of other adverse reactions.

A total of 105 patients in the 12 studies^13^^,^^16^^,^^18^^,^^21^, ^22^, ^23^, ^24^^,^^27^, ^28^, ^29^, ^30^ of 1374 patients reported abdominal pain after taking the drug. There was no statistical heterogeneity between the studies (p = 0.921, *I^2^* = 0.0%). After a fixed effect model analysis, the results showed that the difference between the test group and the control group was statistically significant (OR = 1.42, 95% CI 0.94‒2.14, p = 0.094). Taking PEG combined with lactulose showed a lower incidence of abdominal pain compared with taking PEG alone. The forest plot of abdominal pain is shown in Fig. 5A.

### Abdominal distension

A total of 156 patients in the 12 studies^13^^,^^16^^,^^18^^,^^21^, ^22^, ^23^, ^24^^,^^27^, ^28^, ^29^, ^30^ of 1374 patients reported abdominal distention after taking the drug. There was no statistical heterogeneity between the studies (p=0.876, *I^2^* = 0.0%). The results showed no significant difference in the incidence of abdominal distention between the PEG combined lactulose group and the PEG alone group (OR = 1.32, 95% CI 0.94‒1.85, p = 0.114). The forest plot of abdominal distention is shown in Fig. 5B.

### Nausea

A total of 148 patients in the 13 studies^13^^,^^16^^,^^18^^,^^21^, ^22^, ^23^, ^24^, ^25^, ^26^, ^27^, ^28^, ^29^, ^30^ of 1522 patients reported nausea after taking the drug. There was no statistical heterogeneity between the studies (p = 0.839, *I^2^* = 0.0%). After a fixed effect model analysis, the results showed a statistically significant difference in nausea between the test group and the control group (OR = 1.60, 95% CI 1.13‒2.28, p = 0.009). In the PEG combined with the lactulose group, the incidence of nausea was lower compared with the PEG alone group. The forest plot of nausea is shown in Fig. 5C.

### Vomiting

A total of 93 patients in the 10 studies^13^^,^^16^^,^^18^^,^^22^, ^23^, ^24^^,^^27^, ^28^, ^29^, ^30^ of 1284 patients reported vomiting after taking the drug. There was no statistical heterogeneity between the studies (p=0.934, *I^2^*=0.0%). After a fixed effect model analysis, the results showed a statistically significant difference in vomiting between the test group and the control group (OR = 1.77, 95% CI 1.14‒2.74, p = 0.011). In the PEG combined with the lactulose group, the incidence of vomiting was lower compared with the PEG alone group. The forest plot of vomiting is shown in Fig. 5D.

### Other adverse reactions

There were 20 patients who reported other adverse reactions such as dizziness, headache and weakness, from 4 studies^13^^,^^25^^,^^28^^,^^29^ involving 634 patients. No statistical heterogeneity between the studies (p = 0.899, *I^2^* = 0.0%) was observed. After a fixed effect model analysis, the results showed that there was no significant difference in the incidence of other adverse reactions between the PEG combined with lactulose group and the PEG alone group (OR = 2.79, 95% CI 1.02‒7.60, p = 0.045). The forest plot of vomiting is shown in Fig. 5E.

### Publishing bias

**Fig. 6:**
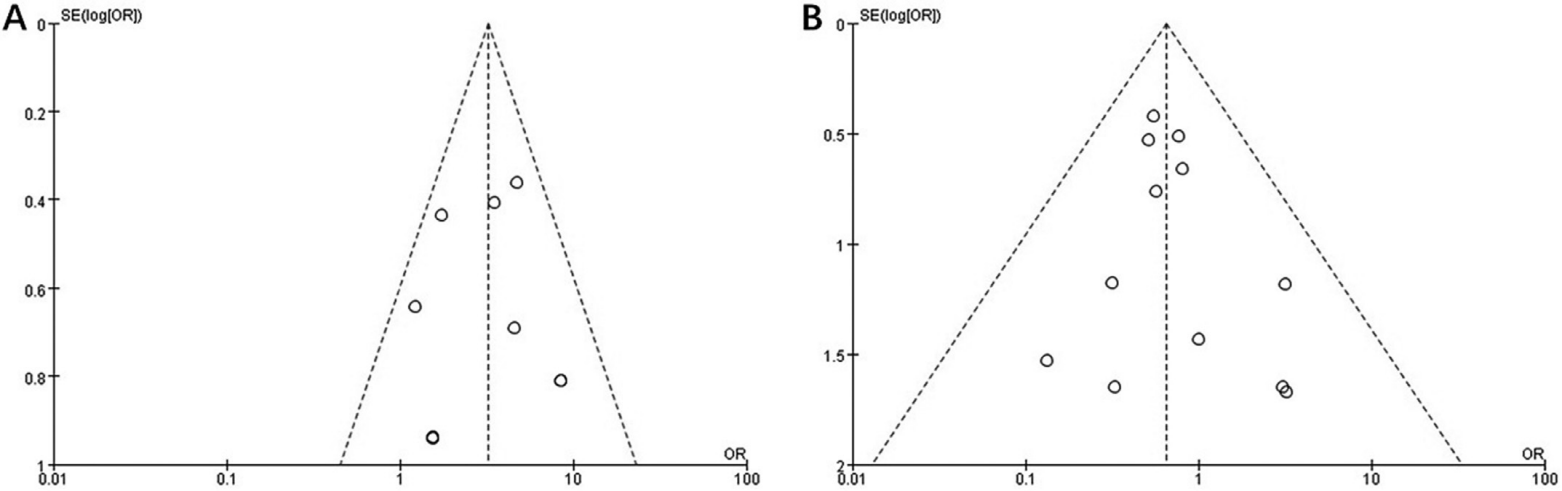
The publishing bias analysis by the funnel plot. (A) Funnel plot of bowel preparation quality. (B) Funnel plot of abdominal pain efficacy.

The authors also carried out the analysis of publishing bias based on the efficiency of bowel preparation and adverse reaction abdominal pain index. The results showed that the funnel chart is symmetrical, indicating no certain publishing bias in the results of the study. The funnel plot is shown in Fig. 6.

## Discussion

The quality of bowel preparation before a colonoscopy is an important determinant of the cecal intubation time, observation of lesions, and detection rate of minor lesions. Perfect bowel cleansing makes it easier to detect macroscopic lesions in the colon and terminal ileum.^31^ The ideal bowel preparation should be effective, safe, economical, and well-tolerated.^32^ PEG is widely used in bowel preparation, but the effect of using PEG alone in intestinal cleansing is often unsatisfactory. Lactulose is a palatable laxative that can stimulate the peristalsis of the colon and restore the normal physiological rhythm. It causes few adverse reactions and boasts a positive effect on the relief of constipation symptoms. Therefore, many researchers suggested that combining PEG and lactulose for bowel preparation might be more effective.

In the study, the authors systematically assessed the quality of bowel preparation by PEG combined with lactulose compared with PEG alone. According to different evaluation standards for the quality of bowel preparation, the authors divided the enrolled studies into two groups. Both evaluation methods showed that PEG combined with lactulose was better than PEG alone for bowel preparation. There was low heterogeneity between the studies observed and the funnel chart showed that the included studies had low publication bias. In the subgroup analysis, there was high heterogeneity between patients without constipation in the efficiency group. It may be due to the different evaluation standards and the small number of documents included.

Patients with constipation have poor intestinal drainage function, which is an important factor that affects the quality of bowel preparation. In clinical practice, the quality of bowel preparation in patients with constipation can often be improved by increasing the amount of compound PEG dispersion to 4000 mL. Nevertheless, many patients are reportedly subjected to poor bowel preparation quality due to chronic constipation.^33^ Therefore, the authors performed a subgroup analysis to determine whether PEG combined with lactulose was more effective in patients with constipation. The results showed that in patients with constipation, the usage of PEG combined with lactulose as the bowel preparation medication had a better cleansing effect than PEG alone. PEG combined with lactulose may be a better option to improve the quality of bowel preparation in constipation patients.

The occurrence of adverse reactions after drug administration often affects the compliance of patients, which in turn influences the quality of bowel preparation. The meta-analysis showed that PEG combined with lactulose had fewer adverse reactions, including abdominal pain, nausea, and vomiting, than PEG alone. No significant reduction in the incidence of abdominal distention was observed, which might be caused by the large dosage of PEG. However, the authors did not find any study that compares the effects of a regular dose (3‒4 L) with a low dose (1‒2 L) of PEG combined with lactulose in bowel preparation. If feasible, the authors will do further research to investigate whether a low dose (1‒2 L) of PEG combined with lactulose has a better effect on bowel cleansing compared with a high dose (3‒4 L) alone.

This study is the first comprehensive analysis that compares the efficacy of PEG combined with lactulose with that of PEG alone in bowel preparation before colonoscopy. However, there are several limitations. First, nearly 90 percent of the included articles were conducted in the Chinese population. Considering regional differences, the results above are probably more applicable to Asians. Second, the PEG dosage was not the same, and the small number of included studies did not allow a further subgroup analysis. In addition, several studies indicated that PEG combined with other drugs such as magnesium sulfate or gastrointestinal prokinetic drug could also improve the bowel cleansing effect.^17^^,^^20^ Whether lactulose is a better choice compared with other drugs requires further investigation.

## Conclusion

PEG combined with lactulose could improve bowel cleansing effect with fewer adverse reactions, thus serving as a simple, convenient, safe and effective method for bowel preparation. As the optimal administration method has not been clearly defined, large and well-designed cohort studies need to be carried out.

## Ethics approval and consent to participate

Not applicable.

## Consent for publication

Not applicable.

### Availability of data and materials

All relevant data for this study are presented in tables and figures.

## Authors' contributions

XFZ designed the study and wrote the manuscript. YSC did the critical revision of the manuscript. WZ conducted a systematic literature search. CXT performed the literature review and data abstraction. YC and SL analyzed data. LL is the study supervisor. All authors have read and approved the manuscript.

## Funding

No funding was received for this study

## Conflicts of interest

The authors declare no conflicts of interest.
